# P02.165. A comparative effectiveness trial of high-quality vitamin D3 nutritional supplements to replete serum vitamin D

**DOI:** 10.1186/1472-6882-12-S1-P221

**Published:** 2012-06-12

**Authors:** M Traub, R Bradley, J Finnell

**Affiliations:** 1 Lokahi Health Center, Kailua Kona, USA; 2 Bastyr University, Kenmore, USA

## Purpose

The primary aim is to compare the change in serum 25-OHD concentration between three forms of supplemental vitamin D3: a lipid-emulsified form administered in a sesame oil base, a non-emulsified chewable tablet, and a non-emulsified form administered to 25-hydroxycholecalciferol (25-OHD) insufficient <33ng/ml (75 nmol/ml) patients. The secondary aim is to compare the proportion of participants reaching an “optimal” 25-OHD concentration ≥33ng/ml (75 nmol/ml) between Vitamin D supplement groups following supplementation.

## Methods

This three-arm, randomized clinical trial compared the difference in serum 25-OHD concentration between the three arms at baseline and after random administration of one of the three vitamin D preparations for 12-weeks at a dosage of 10,000 IU Vitamin D per day (N=60 vitamin D insufficient subjects and N=30 sufficient controls).

## Results

Enrollment occurred from August 2010 to August 2011. Vitamin D insufficient, age-eligible participants were enrolled in the study (N=66), with the addition of sufficient controls (n=37). Overall loss to follow up was n=11 (16.7%). Between group differences were not significant for age, gender, height, weight or baseline Vitamin D 25 status. One-way analysis of variance (ANOVA) was used to estimate the difference in between group means. The mean unadjusted intra-group increase in serum 25-OHD were: Group A, 33.3 ng/mL (95%CI 24.1 – 42.4); Group B, 33.5 ng/mL (95%CI 19.6 – 47.4 ng/mL); Group C, 53.6 (95%CI 40.7 – 66.4). The serum levels of 25OHD were significantly different between groups (p=0.0215), while the serum levels of 1,25-OH2D did not reach between group significance (p=0.4850). The proportion of participants reaching 25-OHD ≥33ng/ml were: Group A 100%; Group B 82%; and Group C 100%.

## Conclusion

Between group differences reached significance for mean change in 25-OHD status. Final analysis of results will adjust for confounding, after which the treatment arm assignments will be un-blinded. Analysis of cardio-metabolic data, Klotho protein expression and TLR-4 expression are in process.

